# Nickel Wall-coated Microreactors for Enhanced Sensitivity and Peak Resolution in Compound-specific Carbon and Nitrogen Isotope Analysis

**DOI:** 10.1021/acs.analchem.6c00746

**Published:** 2026-05-18

**Authors:** Habib Al-Ghoul, Martin Elsner

**Affiliations:** Technical University of Munich, TUM School of Natural Sciences, Department of Chemistry, Chair of Analytical Chemistry and Water Chemistry,Lichtenbergstraße 4, 85748 Garching, Germany

## Abstract

Online combustion of analytes through gas chromatography-combustion-isotope ratio mass spectrometry (GC-C-IRMS) has facilitated compound-specific isotope analysis (CSIA) for various applications like tracing environmental contamination and doping in sports. However, GC-C-IRMS critically depends on complete peak separation, where current combustion tubes with an inner diameter (i.d.) of 0.5 mm and threaded nickel wires are a notorious source of peak broadening. To reduce the reactor volume by 3–6 fold, we instead produced Ni wall-coatings in catalytic microreactors of alumina and quartz (0.2 mm–0.3 mm i.d.). Electroless plating coated the tube wall over a length of 18–20 cm covering the hot zone of the furnace. Coating thickness, characterized by scanning electron microscopy, was (1.23 ± 0.12 μm, *n* = 7) and the total mass of deposited Ni was 0.10 mg/cm. A seed oxidation of 2 min before each chromatographic run facilitated accurate carbon and nitrogen isotope analysis of caffeine: Δδ^13^C = −0.16‰ ± 0.12‰ (quartz capillary, i.d. 0.30 mm, splitless injection, *n* = 232); Δδ^13^C of 0.05‰ ± 0.09‰ and −0.16‰ ± 0.13‰ (two replicate alumina reactors, i.d. 0.20 mm, split injection, *n* = 336 and 347 respectively) and Δδ^15^N = 0.23‰ ± 0.22‰ (alumina reactor, i.d. 0.20 mm, split injection, *n* = 29). Increasing the residence time in the reactor enabled even accurate isotope analysis of atrazine (Δδ^13^C = 0.03‰ ± 0.21‰, n = 30; Δδ^15^N = −0.05‰ ± 0.15‰, *n* = 15) as a structure notoriously resistant to complete oxidation. A proof-of-principle test with *n*-alkanes demonstrated a 10-fold higher sensitivity and 2-times better peak resolution compared to the current commercial configuration. Hence, our setup paves the path to developments for which sensitivity and peak width are critical like environmental trace analysis and comprehensive two-dimensional gas chromatography (GCxGC-C-IRMS).

## Graphical abstract

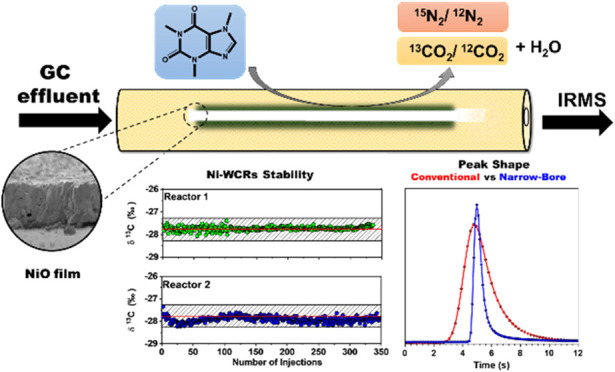

## Introduction

High-precision isotope ratio mass spectrometry (IRMS) is uniquely suited to accomplish precise measurements of isotope ratios of different elements C, N, O, S, Cl, and H at their natural isotopic abundance. For measurements, organic samples are converted into small molecules, such as CO_2_, N_2_, O_2_, SO_2_, or H_2_.^1^ To accomplish such quantitative transformation online, the continuous carrier gas stream that exits from a gas chromatographic system is channeled into a miniaturized reactor tube in a high-temperature conversion interface.^1^ For carbon isotope analysis, oxidative transformation produces CO_2_, which is the common measurement gas for analysis of carbon isotope ratios (R = ^13^C/^12^C), whereas N_2_ is the measurement gas for nitrogen isotope analysis (R = ^15^N/^14^N). By comparing the isotope ratio of the sample to that of a traceable reference standard, this setup enables the determination of slight variations in stable isotopic abundances using IRMS in permille (‰) in the delta notation (δ)­(eq 1).^2^

1 $$\delta^{13}C=\frac{R_{\mathrm{samp}le}}{R_{\mathrm{reference}}}-1$$

Compared to “traditional” bulk IRMS where whole organic samples are offline combusted to CO_2_, this online combustion interface between gas chromatography and isotope ratio mass spectrometry (GC-C-IRMS, see Figure 1a) has been a dramatic innovation because it has enabled compound-specific isotope analysis (CSIA), that is, the measurement of isotope ratios in single organic substances.^3^ After separation by gas chromatography, the organic compounds are converted within the carrier gas stream so that chromatographic resolution is preserved to a large extent and isotope ratios can be attributed to the respective compounds by their retention time. Isotope ratios determined by CSIA are used, for example, as the equivalent of forensic fingerprints to determine the origin of specific substances and their geographic location. Applications include the detection of steroid doping in sports^4^ and the authentication of foods,^5^ natural products, environmental pollutants,^1^ and petrochemicals.^6^

**Figure 1:**
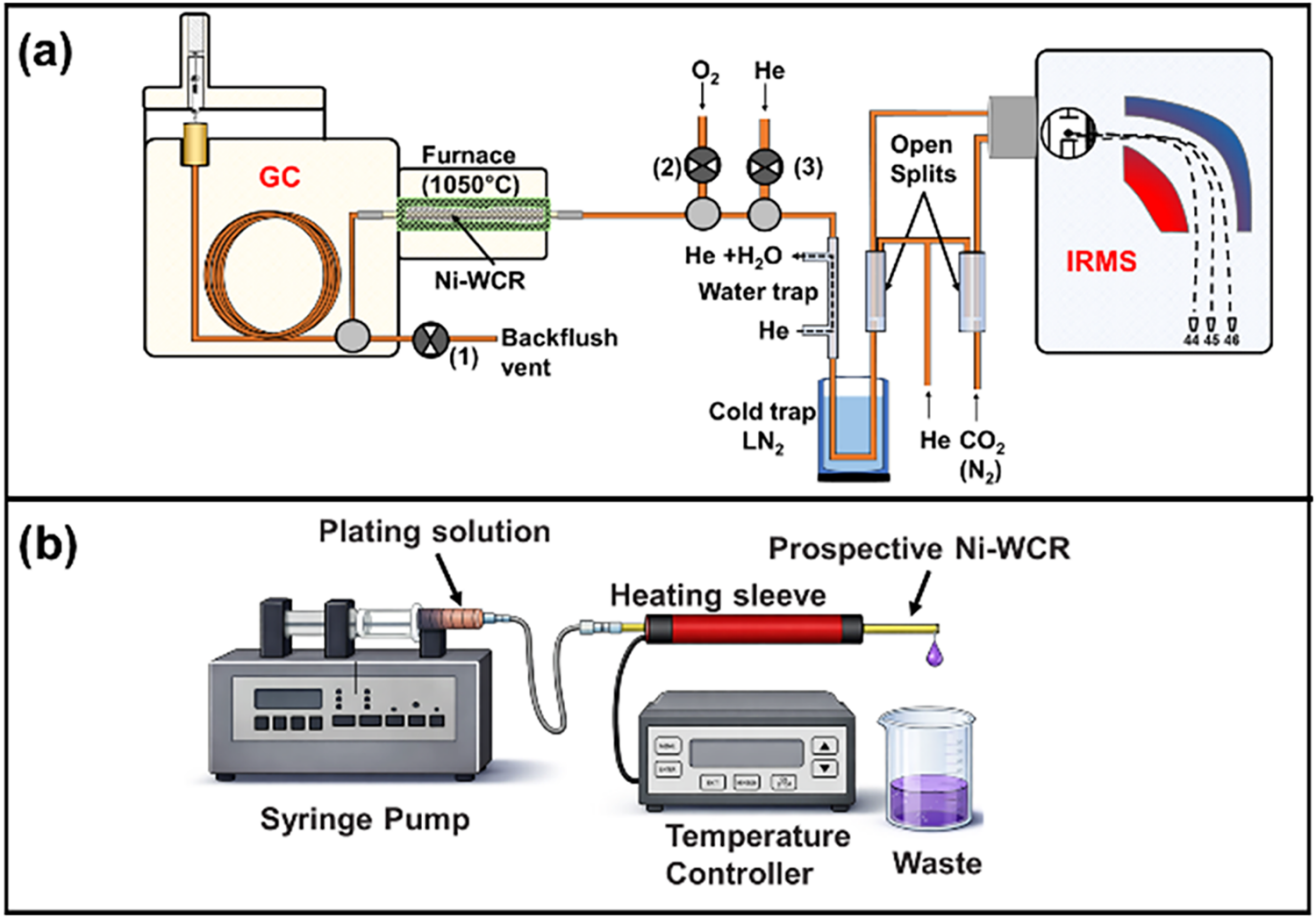
(a) Schematic representation of a GC-C-IRMS system for carbon and nitrogen isotope analysis. In backflush mode, a reversed helium flow from valve 3 is used to divert the effluent from the GC through valve 1 to blend out solvent peaks. When opening valve 2, this mode can also serve to introduce oxygen into the reactor for reoxidation. When all valves are closed (straight mode), the GC effluent reaches the open split, where part of it (0.4 mL/min) is directed to the IRMS, while the rest is vented out by a continuous He stream, as explained in more detail in Figure 5. (b) Schematic of the apparatus used for flow electroless plating of Ni onto a thick wall capillary.

Current combustion interfaces for oxidation of analytes in GC–IRMS consist of nickel wires in a ceramic tube,^3^ or in a ceramic-supported nickel tube^7^ ranging in length from 150 to 200 mm^1^ and heated between 850 and 1050 °C. The first-generation of commercial oxidation reactors for GC–C–IRMS has consisted of ceramic tubes with an inner diameter of 0.5 mm with Cu, Ni, and Pt wires inserted inside.^7^ Before sample analysis, Cu and Ni wires are oxidized by passing O_2_ through the reactor. Subsequently, the CuO and NiO surfaces act as a source of O_2_ in conjunction with a Pt catalyst to facilitate complete combustion. Since incomplete oxidation^3^ may lead to isotope fractionation and peak tailing in the GC effluent, the wires should be placed within the furnace’s hot zone, because at temperatures under 850 °C, catalytic surfaces can present active sites for deposition, preventing complete oxidation.^8^

A prominent downside of current commercial combustion tubes is that their diameter is significantly wider than that of typical gas chromatographic columns, which presently causes turbulences and peak broadening. Broader peaks enable less chromatographic resolution, which can lead to inaccurate results. A minimum resolution of 1.5 is required for CSIA^5^ because of a phenomenon known as the chromatographic isotope effect. ^12^C isotopologues of the analyte typically migrate through the GC column slightly slower than ^13^C isotopologues so that isotopes are not uniformly distributed over the chromatographic peak.^9^ In the case of peak overlap, this makes representative integration of peaks impossible. Moreover, since the sensitivity of IRMS is determined by the peak height rather than the peak area, broadening the same analyte mass into a wider and less intense peak will increase the sample amount required. The problem is emphasized by the low natural abundance of ^13^C. Counting statistics according to the shot noise limit imply that precise ^13^C/^12^C analysis requires a hundred times more analyte than analysis by conventional GC-MS if all other parameters are the same. This means that large volumes of natural samples must be extracted, leading to coextraction of interfering compounds and exacerbating challenges for peak separation. Hence, there is an evident need for combustion reactor tubes with a smaller diameter that minimizes peak broadening, leads to sharper and higher peaks, and preserves peak separation.

Such narrower reactor tubes imply also the use of narrower GC columns with lower volumetric flows. Presently, helium flow rates in commercial GC-C-IRMS instruments—and in connected combustion interfaces—are typically around 1.4 mL/min and should not be decreased, or otherwise peak broadening would become worse. This has two consequences. First, the flow that can be channeled into the ion source of the IRMS is only 0.4 mL/min, since otherwise the high vacuum inside the IRMS would be compromised. Hence, the surplus flow of 1 mL/min is routinely vented via an open split (see Figure 1a) meaning that the major part of applied analytes presently gets lost and sensitivity is by default reduced by a factor of three to four.^10^ Second, such high flow rates of the carrier gas imply short residence times of the analyte in the combustion interface. It is the opposite, however—smaller flow rates—that would be expected to benefit sensitivity and accuracy because higher residence times may enhance conversion efficiency.

To reduce reactor volumes and associated flows, previous studies brought forward a miniaturized design in which a 0.22–0.32 mm deactivated fused silica (FS) capillary was used instead of the ceramic tube.^11–13^ A single or double strand of 0.1 mm metal wire (Ni or Cu) was introduced into the capillary and oxidation equivalents were provided by a constant supply of O_2_ in the form of a trickling stream of oxygen in the carrier gas.^12^ The 0.22 mm internal diameter (ID) corresponds to a volume decrease of 89% compared to 0.65 mm ID, the current ceramic tube dimensions measured by SEM.^14^ At the same time dead-volume connections were eliminated in the rest of the GC-C-IRMS instrument to decrease the possibility of peak broadening and leaks within the connector system.^14^ While the reported measurement performance of the setup has been promising, a prominent weakness has been its robustness and longevity. In particular the manual threading of wires into the microscopic capillary demands experience and patience, as any error could compromise the entire system. Further, the polyimide coating at the outside of the capillary gradually decomposes at high operating temperatures, reducing its mechanical strength so that capillaries are easy to break, especially when wires touch the wall of the capillary from the inside. Consequently, Nickel-wire capillary reactors are reported to have a limited longevity of around one month or one hundred runs.^13^

In order to improve the physical stability of the reactor, Tobias et al. fabricated high-purity fused silica microreactors with semicircular cross-section microchannels of varied widths (56–209 μm) and tapered regions connecting to input/output ports (>400 μm) for fused silica capillary tubing.^15^ A CuO/NiO (55%/45%) wire was threaded into the tubing as combustion source at 1150 °C with 1% O_2_ added to the carrier gas. When hyphenated to fast-GC-C-IRMS, these microreactors provided carbon isotope ratio measurements of methane with a precision of δ^13^C (SD) 0.28‰. However, the restriction of the flow to narrow capillaries at high temperatures led to peak broadening: according to the ideal gas law, a temperature increase from 300 °C (GC temperature) to 1150 °C (temperature of the hot zone in the reactor) will lead to a volume expansion of 3.6. If the volume is restricted and expansion cannot take place, the pressure increases leading to an increased gas viscosity and lower flow rates. Countering this effect by raising the pressure is not an option: rates would have to be increased to 3–6 mL/min, which significantly decreases sensitivity. Consequently, capillary and reactor tube dimensions must be matched such that the inner diameter of the reactor should be approximately 1.5 times larger than of the GC-capillary. For fast-GC, which requires narrow-bore columns with diameters ranging from 0.1 to 0.18 mm, the recommended inner diameter of the reactor would thus fall into the range between 0.15 and 0.3 mm.

Besides their limited longevity and the compromise between sensitivity and peak resolution, the reactors in available publications have only been evaluated by their ability to serve for carbon isotopes analysis. However, isotope analysis of multiple elements is crucial for many applications such as tracing the degradation of chemical micropollutants.^16^ Specifically, to enable carbon isotope analysis, all reported miniaturized reactor designs to date have involved a trickling stream of oxygen in the carrier gas in order to achieve complete combustion to CO_2_. However, this makes the analysis of nitrogen-containing compounds problematic. The permanent introduction of O_2_ will impact the reactor’s ability to reduce nitrogen oxides (NO_*x*_). The incomplete reduction of NO_*x*_ to N_2_, in turn, affects not only δ^15^N but also δ^13^C due to interference at *m*/*z* 46.

In summary, the field is still awaiting a breakthrough in the reactor tube design that would provide the basis for enabling fast GC-C-IRMS and GCxGC-C-IRMS on a routine basis. Specifically, much-needed developments in GC-C-IRMS—such as analysis of low concentrations, of samples with background interferences, and the prospect of comprehensive two-dimensional gas chromatography (GCxGC)—hinge on (i) the development of robust miniaturized combustion tubes and (ii) solutions to the trade-off between peak resolution and sensitivity. Such targeted reactors

i. should carry the catalyst specifically in the hot zone of the furnace to avoid incomplete combustion and peak tailing.
ii. must be sufficiently narrow to align with the GC column to prevent peak broadening and to preserve the advantages of fast GC-C-IRMS and GCxGC-C-IRMS techniques.
iii. should, at the same time, be wide enough to allow for low gas flow rates without restriction at elevated temperatures and should possess an adequate surface area and oxidation capacity to ensure the complete conversion of multiple target analytes.
iv. must be constructed from a rugged, temperature-stable, nonporous material.
v. must accommodate adequate flow rates to ensure sufficient reaction time for full conversion in the oven.
vi. should facilitate the removal of oxygen from nitrogen oxides (NO_*x*_) to enable also the analysis of nitrogen-containing compounds.

Given that the metallic oxide surface facilitates the combustion reaction, there is a need for an alternative design to the traditional bulky wires. In this context, a wall-coated reactor (WCR) has the potential to be a game changer. By coating the inner walls of the reactor with a catalytic metal, sufficient surface area for oxidation may be provided while utilizing a more compact tube size. Nickel is a promising candidate for the last requirement, eliminating the need for a separate reduction reactor so that it downsizes the potential dead volumes and connections in the system (Figure 1a).^7,17^ However, it must operate above 1050 °C, necessitating an internal diameter of 0.2 to 0.32 mm to meet the other criteria.

Consequently, the first objective of this study was to design and evaluate nickel wall-coated reactors for the analysis of δ^13^C and δ^15^N isotope values by electroless plating. Our second aim was to test various substrates and to assess the oxidation capacity, linearity, and longevity. Our third objective was to specifically test the ability of the reactors to remove NO_*x*_ and to enable accurate ^15^N measurements. Further, we paid particular attention to the optimum flow rate for effective conversion. Given that peak amplitudes depend on an intricate relationship between flow rate, residence time in the conversion reactor and dilution in the open split, we examined the effect of dilution independently by measuring the response of argon as an inert gas. Finally, we provided a proof-of-principle by conducting a test run in which we aimed to eliminate all other sources of peak broadening (water trap, transfer lines, open split, etc.) in the commercial instrumental setup.

To validate the accuracy of isotope analysis in our reactors, we selected, besides higher chain alkanes, compounds that contain carbon and nitrogen: starting with caffeine, which is commonly used to test and validate newly installed reactors;^18^ continuing with atrazine, which has been found to be particularly challenging to analyze;^7^ and finally 1H-benzotriazole, which is reported to be retained at the surface of some metals so that it is not detectable by conversion in Cu/Ni/Pt-containing oxidation reactors.^19^

## Materials and Methods

### Chemicals and Materials

The international isotope standards of caffeine were IAEA 600 (International Atomic Energy Agency, Austria), δ^13^C_IAEA 600_ = −27.771‰ and δ^15^N_IAEA 600_ = 1.0‰, and USGS62 (U.S. Geological Survey, USA), δ^13^C_USGS62_ = −14.79‰ and δ^15^N_USGS62_ = +20.17‰.^18^ Atrazine (1-chloro-3-ethylamino-5-isopropylamino-2,4,6-triazine, 97.7%,CAS: 1912–24–9) was supplied by Tropitzsch (Marktredwitz, Germany). It was our in-house standard IS 29, whose isotopic composition had been previously characterized as δ^13^C_IS 29_ = −28.59‰ and δ^15^N_IS 29_ = −1.3‰ using an elemental analyzer (EA, Carlo Erba) coupled with an isotopic ratio mass spectrometer (IRMS).^7^ Undecane (*n*-C_11,_ ≥ 99.0%), dodecane (*n*-C_12_, ≥ 99.0%), tridecane (*n*-C_13_, ≥ 99.0%), tetradecane (*n*-C_14_, ≥ 99.0%), pentadecane (*n*-C_15_, ≥ 99.0%), hexadecane (*n*-C_16_) (99%), benzotriazole (99%), methanol (pestanal, 99.9%), nickel­(II) acetate (≥99.0%), ammonium hydroxide solution (25%), hydrazine (34%), formaldehyde (35%), and nitric acid (70%) were from Merck, Darmstadt (Germany). Table 1 shows the tubes used in this study and their properties.

**Table 1:** Properties of the Thick Wall Capillaries Used to Produce Reactor Tubes

material	inner diameter (mm)	outer diameter (mm)	length (mm)	tube volume (μL)	surface area^a^ (mm^2^)	manufacturer
quartz	0.3	1.5	305	21.5	188.6	CM Scientific Ryefield (EU) Ltd. (Dublin, Ireland)
quartz	0.28	1.5	320^b^	19.7	176.1	QSIL GmbH Quarzschmelze (Ilmenau, Germany)
alumina	0.2	1.5	320	10.0	125.7	KYOCERA Fine Ceramics Europe GmbH (Mannheim, Germany)
alumina (conventional design)	0.5	1.5	320	62.8^c^	188.6^d^	KYOCERA Fine Ceramics Europe GmbH (Mannheim, Germany)

a Surface Area of the Coating Applied within the Corresponding Length of the Hot Zone (200 mm).

b The length of the commercial tube was originally 360 mm but cut to 320 mm in our study for comparability.

c Tube Size of Conventional Reactors (with Metal Wires, Not Coated).

d Surface Area of Three Threaded Wires (No Coating).

### Instrumentation

A GC-C-IRMS system consisting of a TRACE GC Ultra gas chromatograph (Thermo Fisher Scientific, Milan, Italy) coupled to a Finnigan MAT 253 isotope ratio mass spectrometer (IRMS) (Thermo Fisher Scientific, Bremen, Germany) via a Finnigan GC Combustion III interface (Thermo Fisher Scientific) was used for measurements. The reduction oven tube of the commercial setup was eliminated to study the performance of Ni wall-coated reactors (Ni-WCRs) in the measurement of nitrogen-containing compounds (see Figure 1a). For nitrogen isotope measurements a liquid nitrogen (LN_2_) trap was positioned between the combustion interface and the IRMS to freeze the carbon dioxide generated during the combustion of the analyte. This measure ensures that carbon dioxide does not enter the ion source, thus preventing any potential interference from CO^+^ in the measurement of N_2_^+^ isotopologues. The collected carbon dioxide was released from the trap daily by warming to room temperature. The emission current was set to 2.0 mA. Helium (grade 5.0) was used as a carrier gas, and liquid samples were injected via a GC Pal autosampler (CTC, Zwingen, Switzerland). The analytical column was a Rtx-xil-5 ms (20 m_0.18 mm; 0.36 μm film; Restek). Isotope ratios were measured against a laboratory monitoring gas (CO_2_ and N_2_) before and after each run, which in turn was calibrated against international reference standards USGS-61, USGS-62, and USGS-63 for carbon. The same standards were used for nitrogen reference gas calibration plus air and IAEA-600.

For our proof-of-principle demonstration of narrower peak shape, we further eliminated additional sources of peak broadening by replacing the transfer lines, the Nafion water trap, and the open split from the commercial setup with zero-dead-volume-alternatives for a limited number of supervised injections. The system was equipped with an AS-5 MS Xil FAST GC column (15 m_0.10 mm; 0.1 μm film; Altmann) in a TRACE GC Ultra gas chromatograph connected with narrow-bore capillaries, including Ni-WCR, with a 0.6 mL/min carrier gas flow rate. While further optimization is ongoing to convert this optimized setup into a robust instrument configuration for routine analysis, this development is beyond the scope of the current study.

The peak shape and sensitivity of this optimized setup was compared for identical conditions (injection of 0.5 μL of an *n*-alkane mixture with a 100:1 split ratio at a temperature of 280 °C) into a state-of-the-art commercial system composed of a Trace 1310 gas chromatograph (Thermo Fisher Scientific, Germany) equipped with a J&W DB-5MS UI column (30 m × 0.25 mm, 1.0 μm film thickness, Agilent) operated at a column flow rate of 1.4 mL/min and connected to a MAT 253 stable isotope ratio mass spectrometer via an GC-IsoLink interface (all devices from Thermo Fisher Scientific, Germany).

### Reactor Fabrication

We employed flow electroless plating to coat the inner capillary wall with nickel. This autocatalytic process allows metal deposition on substrates such as ceramics and glass. Electroless plating is notable for its ability to coat various metal catalysts, which is achieved by selecting an appropriate plating solution.^20^ Figure 1b shows the setup to coat reactor tubes with Ni. A syringe pump (*K*_d_ Scientific, MA, USA) was used to introduce the coating solution into the reactor at a constant flow. A heating sleeve with the corresponding heating unit MonoSLEEVE (Analytical Sales and Services, Inc., NJ, USA) covering a length of 17 cm and a diameter of 1/16” was used for temperature control to enable the coating of only a specific section of the capillary which would later correspond to the hot zone of the oven. Initially, 24 mL of nickel acetate (NiAc_2_) solution (0.433 g, 1.74 mmol) was sonicated for 7 min at room temperature to liberate O_2_ from the solution. The tube was pretreated by flushing a concentrated ammonium hydroxide solution (25%) for 5 min. Subsequently, the plating solution was prepared by adding 1 mL of hydrazine (35%) and two drops of formaldehyde (37%) to the nickel acetate solution resulting in a color change from turquoise to dark blue.^21^ The tube was connected to a syringe containing the plating solution and then fit into the heating sleeve. The heater was adjusted to 95 °C, and the tube was coated for 30 min at a controlled flow rate of 0.15 mL/min. Under these conditions a coated length of 18–20 cm was achieved. The reactor was then cleaned by flushing with water for 15 min, followed by methanol for another 15 min at room temperature, and dried with nitrogen for 15 min. It was initially annealed for 4 h at 500 °C and then oxidized for 2 h at 1050 °C.

### Characterization

To determine the deposited mass of nickel (Ni) in the capillary, the layer was removed by dissolution in 35% nitric acid, flushing it into a 100 mL volumetric flask and adding deionized water to a defined volume. The amount of nickel was subsequently analyzed by inductively coupled plasma–mass spectrometry (ICP-MS) on a NexIONTM350D ICP-mass spectrometer from PerkinElmer. From these data, the average thickness (t*)* of the layer was calculated by eq ([Disp-formula eq2]), where (*m*) is the mass of nickel, (ρ) is its density, *L* is the deposition length, and *r*_o_ inner radius of the reactor tube

2 $$m=\rho \pi L\left(2r_{o}t-t^{2}\right)$$

In parallel, scanning electron microscopy with energy-dispersive detection of X-ray fluorescence radiation (SEM-EDX, Sigma 300 VP from Zeiss) was utilized to assess the layer thickness and the precise inner diameter of the channel (Figure S1).

### Reactor Performance

Standard solutions in methanol containing the selected compounds were analyzed using the following temperature programs. For carbon isotope analysis, measurements for atrazine, n-hexadecane, and caffeine began at 120 °C (held for 1 min). Then the temperature was ramped at 22 °C/min to 250 °C, followed by a ramp of 40 °C/min to 320 °C (held for 4 min). For nitrogen isotope analysis, the GC program was set to start at 80 °C (held for 1 min) and ramped at 40 °C/min to a final temperature of 310 °C (held for 5 min). The measurements were conducted at various split ratios and column flows.

The performance of carbon and nitrogen isotope ratio measurements using Ni-WCRs was evaluated based on the concentration of the injected target compounds. Lower limits of precise isotope analysis were established to determine the minimum analyte concentration at which the isotope values fell within predefined intervals that accounted for the total analytical uncertainty. Typically, the total analytical uncertainty (corresponding to 2σ) of Compound-Specific Isotope Analysis (CSIA) is ± 0.5‰ for δ^13^C measurements and ± 1‰ for δ^15^N measurements.^10,19,22^ The shifts in isotope values are reported in ‰, indicating the deviation (Δδ^13^C and Δδ^15^N) between the measured isotope values in the processed samples (δ^13^C_measured_ and δ^15^N_measured_) and the respective certified values (δ^13^C_Standard_ and δ^15^N_Standard_), as represented in eqs 3 and 4.

3 $$\Delta \delta^{13}C=\delta^{13}C_{\mathrm{measured}}-\delta^{13}C_{\mathrm{Standard}}$$

4 $$\Delta \delta^{15}N=\delta^{15}N_{\mathrm{measured}}-\delta^{15}N_{\mathrm{Standard}}$$

To investigate the accuracy of carbon and nitrogen isotope values, the lower limits of precise isotope analysis, and the longevity of reactor tube performance with two independently fabricated microreactors of different materials, we analyzed C- and N-containing standards using (a) two Ni-coated alumina capillaries of 0.2 mm inner diameter (Ni-0.20ID-Alumina) and (b) a Ni-coated quartz capillary of 0.3 mm inner diameter (Ni-0.30ID-Quartz). For these measurements, we used international caffeine standards, IAEA 600 and USGS62 plus our in-house standard of atrazine. In addition, n-hexadecane and 1H-benzotriazole were characterized against the international caffeine standards.

## Results and Discussion

### Characterization

Images of the fabricated wall-coated microreactors provide a detailed characterization of the Ni deposition (Figure 2). A cross-sectional scanning electron microscopy (SEM) image of the Ni-coated capillary (Figure 2a) illustrates the presence of double layers, consisting of a quartz tube and a thin inner layer of Ni. SEM analysis (see Figure 2b) shows a close-up of the deposited Ni layer on the quartz surface giving an average measured thickness of the coated layer (*t*_m_) of (1.23 ± 0.12 μm, *n* = 7). ICP-MS analysis of the solution extracted from the Ni-0.28ID-Quartz capillary showed that the amount of the coated nickel was 0.10 mg/cm, corresponding to a calculated thickness (*t*_c_) of 1.30 μm (Table S1). This number is in perfect agreement with the t_m_ derived from SEM indicating an excellent uniformity and purity of coating. The energy-dispersive X-ray (EDX) mapping (Figure 2c and 2d) confirms the purity of the Ni-coated layer, revealing that only Ni atoms were bound to the SiO_2_ surface. Additionally, a sporadic interspersion of Ni atoms within the Si atoms indicates percolation and, hence, a strong adhesion of the two layers. After oxidation of the deposited Ni layer, a green nonporous NiO layer formed (Figure 2e) with increased thickness (Figure 2f) compared to the pure Ni layer (Figure 2b). The results show that temperature-controlled, electroless plating resulted in the successful, uniform deposition of a Ni coating that was allocated selectively in the area of the hot zone of the reactor, with a strong adhesion to the inner wall of the support capillary. This microlayer design minimizes the risk of reactor clogging compared with the bulky wires employed in conventional approaches.

**Figure 2:**
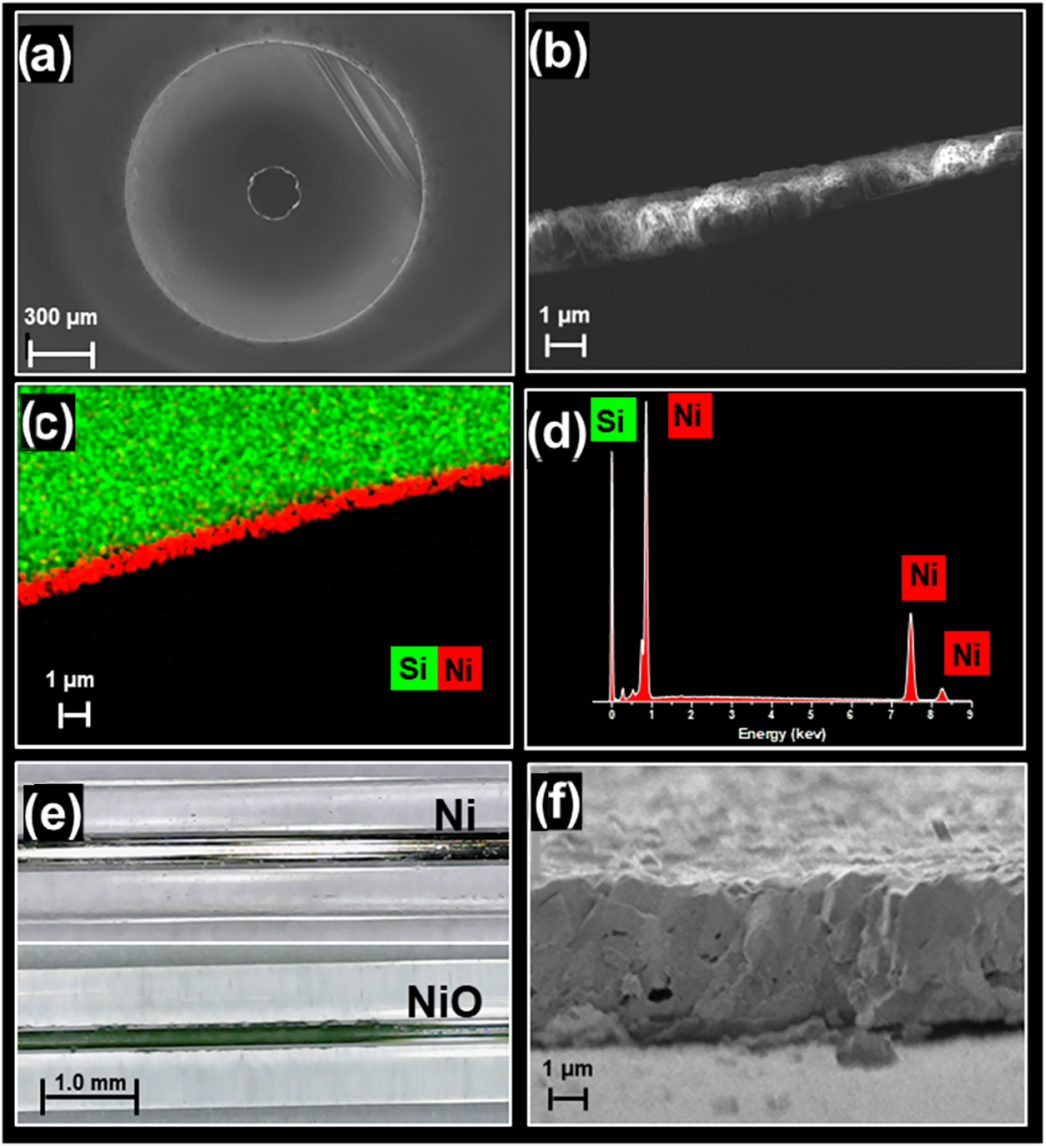
Cross-sectional SEM-EDX images and optical microscope of the capillary microtubes. (a) SEM mapping of the capillary with the nickel layer inside the tube, (b) SEM close-up of the Ni layer, (c) the double layer inner channel of the capillary with EDX mapping, (d) the corresponding EDX spectrum, (e) optical image of the quartz microtubes before and after nickel oxidation, and (f) SEM close-up of the NiO layer after oxidation.

### Carbon Isotope Analysis

#### Oxidation Capacity

An advantage of wall-coated reactors is their high surface-area-to-volume ratio, which offers an extended interface for effective interaction between analytes and catalyst. The void volume of the Ni-0.20ID-Alumina reactor tube is 10 μL, as outlined in Table 1. This volume is equivalent to three wires (single wire size: 0.1 mm OD × 20 cm) housed within a conventional reactor tube of 62.8 μL. The three wires possess a calculated surface area of 188.6 mm^2^. In comparison, the coating accounts for 66% of that surface area (125.7 mm^2^, third entry of Table 1) with a six times smaller void volume than the conventional reactor tube. To fit this oxidation tube within the heater, the outer diameter is the same, 1.5 mm. Hence, the Ni-wall-coated tubes preserve the mechanical stability while allowing a reduction in the inner diameter to minimize void space and prevent peak broadening.

To minimize the formation of nitrogen oxides and to avoid exposure of the mass spectrometer to elevated oxygen levels, reactors were operated without addition of O_2_ (“trickling stream of oxygen”) to the carrier gas flow; instead, the Ni was preoxidized with an oxidation in backflush (0.4 mL/min O_2_ flow) mode so that the thermal dissociation of the NiO provided oxygen for combustion. In fact, NiO does not undergo thermal decomposition spontaneously; instead, it supplies equivalent amounts of oxygen to the organic compounds present in the GC effluent. This effectively mitigates continuous oxygen loss.^3^ As a result, NiO reactors can operate without the need for a long reoxidation every few days. However, a short oxidation with O_2_ “seeding” after each sample run is required to refresh the reactor. Figure 3a demonstrates the effect of this seed oxidation in backflush mode at 1050 °C. When 3 nmol of IAEA 600 were injected into the Ni-0.20ID-Alumina (reactor 1) without seeding, the resultant δ^13^C values deviated significantly, whereas accurate analysis was accomplished with a reoxidation for 2 min following each analytical run.

**Figure 3:**
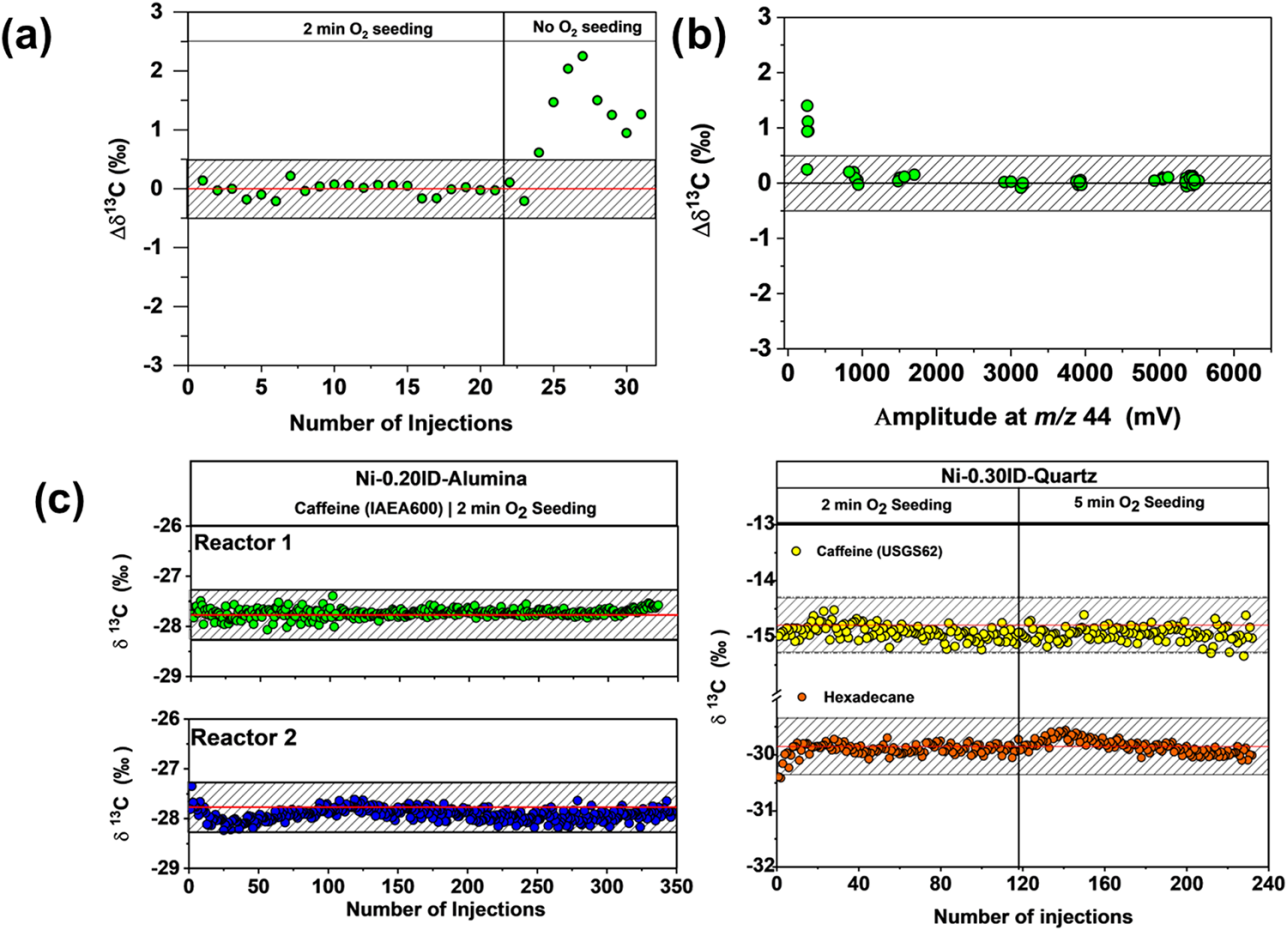
Carbon isotope analysis of caffeine standards. The shaded area represents the total analytical uncertainty (corresponding to 2σ) for carbon Compound-Specific Isotope Analysis (CSIA) of ± 0.5‰. (a) Δδ^13^C values of caffeine (IAEA600) standard with and without O_2_ seeding. (b) Δδ^13^C values for different amplitudes of *m*/*z* 44. (c) Long-term performance of three different combustion reactors (two replicate Ni-0.20ID-alumina reactors, left, and one Ni-0.30ID-Quartz reactor, right) for δ^13^C measurements of different caffeine standards and hexadecane.

#### Linearity

To test for the amount-dependency of isotopic fractionation, caffeine (IAEA 600) was evaluated with injection quantities ranging from 0.6 to 12 nmol C and employing the setup with a Ni-0.20ID-Alumina (reactor 1). The data presented in Figure 3b showed no isotopic fractionation in dependence on the injected amount within this concentration range. With regards to lower limits of precise isotope analysis, Figure 3b demonstrates that a minimum amplitude of 824 mV (*m*/*z* 44) was necessary for precise carbon isotope analysis corresponding to 1.2 nmol of carbon on-column. Figure S2 shows the isotope ratio trace (^13^C/^12^C) for 3 nmol C on-column caffeine analysis. We note that this limit is still relatively high owing to the fact that our narrow-bore reactor was tested under “standard conditions” in a commercial GC-C interface which gave rise to peak broadening due to the width of transfer lines, the Nafion membrane in the water trap, open split, etc.^12^ For a demonstration of the improvement in a fully optimized system, we refer to the section “Conventional versus narrow-bore interface” below.

#### Reactor Stability

Figure 3c presents data from the analysis of caffeine using three different Ni-WCRs, two Ni-0.20ID-Alumina, and Ni-0.30ID-Quartz. The quartz reactor demonstrated accurate δ^13^C values for caffeine USGS62 (−14.95‰ ± 0.13‰, *n* = 232) and n-hexadecane (−29.55‰ ± 0.12‰, *n* = 232) when caffeine was injected alongside with hexadecane in the same chromatographic run. However, the quartz tube could not withstand over two months of operation at 1050 °C. Merritt et al.^3^ noted that quartz, in junction with cupric oxide, can form a eutectic phase, resulting in a rapid failure when exposed to elevated temperatures, which may have occurred with NiO as well.

In comparison, the Ni-0.20ID-Alumina reactors exhibited greater durability and ease of installation, making them suitable for higher-temperature applications. Reactor 1 demonstrated excellent precision and longevity, with over 1000 measurements of different compounds under different conditions, including the setup for nitrogen isotope analysis. Carbon isotope analysis of caffeine IAEA600 resulted in accurate δ^13^C values (−27.71‰ ± 0.09‰, *n* = 336). To further demonstrate the reproducibility of the reactor fabrication, we produced a second reactor (Reactor 2), which was characterized to display a similar accuracy (−27.91‰ ± 0.13‰, *n* = 347).

### Nitrogen Isotope Analysis

NiO-based reactors can also facilitate nitrogen isotope analysis.^17^ To assess the performance of the Ni-WCR, nitrogen isotopic analysis of caffeine and 1H-benzotriazole was conducted with a Ni-0.20ID-alumina (reactor 1) using the same setup as for carbon, with regular seed oxidation and without a trickling stream of oxygen. A representative chromatogram is presented in Figure 4b, which shows a notably low intensity at *m*/*z* 30 (mass of NO^+^), underscoring the effectiveness of the reduction process, thereby eliminating the need for a separate reduction oven. Nitrogen isotope data for caffeine yielded accurate measurements of 1.3‰ ± 0.2‰ (*n* = 29), with a mean offset of 0.3‰ relative to the standard value (1.0 ‰). The results showed excellent linearity, maintaining good precision down to 1.0 nmol N, which corresponded to signals (*m*/*z* 28) of 423 mV (see Figure 4a). Spahr et al. revealed that 1*H*-benzotriazole is particularly challenging to analyze because it was trapped in a conventional Cu/Ni/Pt oxidation reactor, likely due to metal-analyte complexation in colder parts of the system.^19^ In contrast, Figure 4 shows that benzotriazole was confidently detected using a Ni-0.20ID-alumina (reactor 1), in line with previous successful conversion on Ni-surfaces.^19^ The measurements of isotope ratios were precise with an average value of −7.41‰ ± 0.17‰ (*n* = 29) for δ^15^N, with a mean offset of −0.06‰ relative to the standard value (−7.35‰ ± 0.11, *n* = 4) measured by elemental analyzer-IRMS. This study marks the first application of nitrogen isotope analysis using a miniaturized reactor, paving the way also for future rapid compound-specific nitrogen isotope analysis.

**Figure 4:**
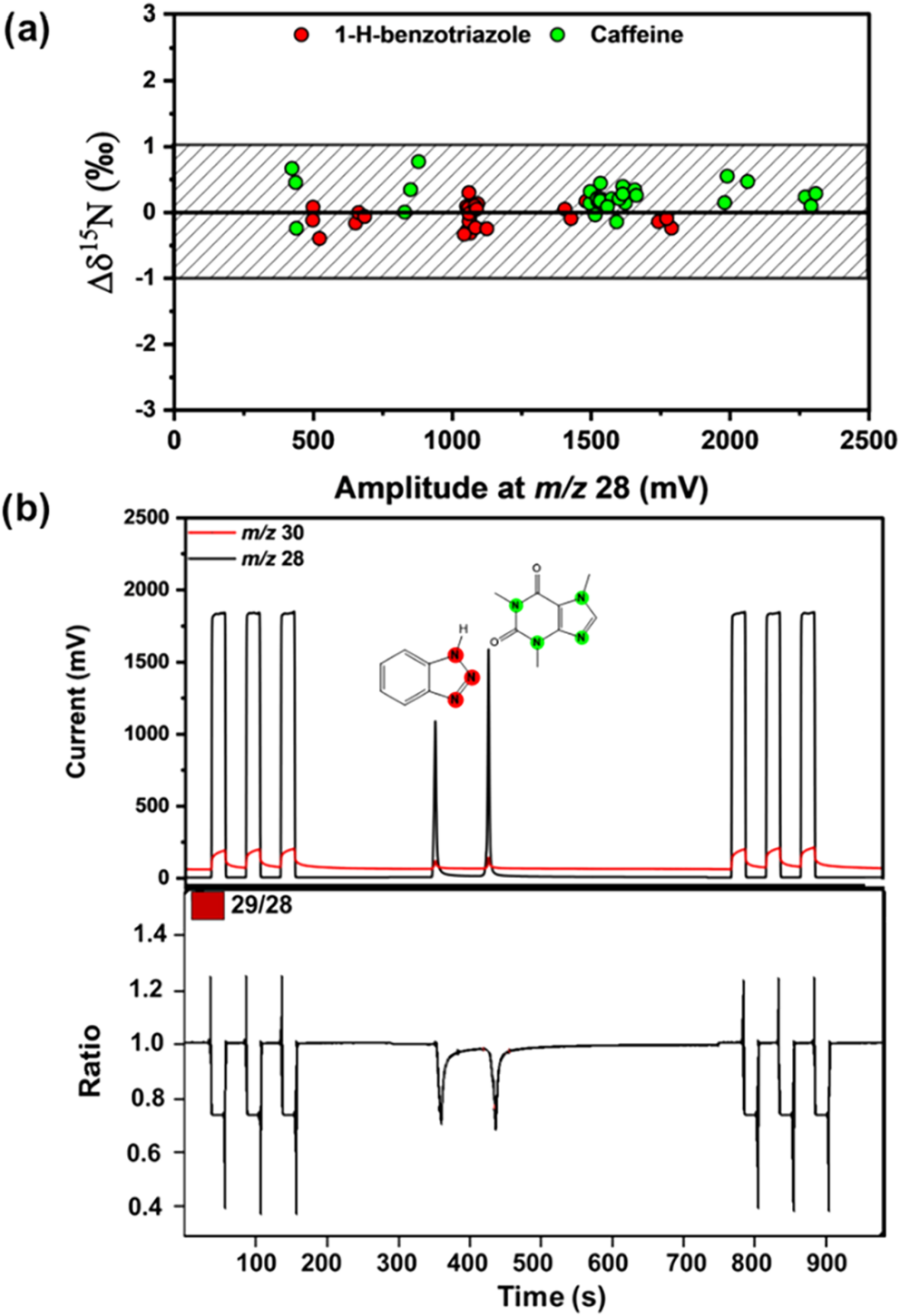
Carbon Isotope analysis of caffeine and 1H-benzotriazole. The shaded area represents the total analytical uncertainty (corresponding to 2σ) for nitrogen Compound-Specific Isotope Analysis of ± 1‰. (a) Δδ^15^N values in dependence on peak amplitudes of *m*/*z* 28 including different injected nitrogen amounts of caffeine (IAEA600) and 1H-benzotriazole. (b) GC-C-IRMS chromatogram for nitrogen isotope analysis (top) with the isotope ratio trace (^15^N/^14^N) (bottom).

### Effect of Column Flow on Conversion Efficiency

#### Dilution and Peak Broadening

The column flow is not only an important factor for chromatographic performance, but it can, in addition, impact the overall sensitivity in multiple ways. On the first hand, depending on the location of the optimum of the van Deemter equation (minimization of longitudinal diffusion vs mass transfer limitations), higher flow rates can sharpen peaks by minimizing diffusion and optimizing peak height (amplitude) and width (effect (i)). In the same way higher flows can minimize longitudinal diffusion in post column capillaries of the interface—where mass transfer to a stationary phase is not of relevance—decreasing the peak broadening (effect (ii)). On the other hand, higher flow rates also mean that a larger sample portion is vented out of the open split in front of the IRMS because the vacuum pumps in the mass spectrometer can only accommodate an inflow of 0.4 mL/min. (Figures 1 and 5) (effect (iii)). Then, higher flow rates also shorten the residence time within the Ni-WCR potentially decreasing conversion efficiency (effect (iv)). Finally, column flow rates may also matter for the geometry of flows in the open split (effect (v)). As illustrated in Figure 5a, the open split in front of the IRMS contains a sniffing capillary which captures 0.4 mL/min of this flow and introduces it into the IRMS. For protection of the IRMS, this split is overlain by a cushion of a continuous He stream. If the column flow becomes small relative to this He stream, potentially, the He cushion may push the column flow out of the open split further diluting the proportion of the analyte that reaches the IRMS. To investigate specifically the effect of column flow on peak shape (effect i and (ii)) and peak dilution in the open split (effects (iii) and (v)), we injected 5 μL of air and analyzed the resultant Argon (Ar) peaks, since Ar serves as an inert analyte that does not undergo conversion in the combustion interface. Figure 5b and c shows the effect of column flow rates on peak area, amplitude and width. As expected, with increasing flow rates, the peak area decreased due to the smaller sample proportion that was channeled to the IRMS (Figure 5b), referred to as the dilution factor. The peak amplitude, however, rose nonetheless (Figure 5b) due to a smaller peak width reflecting the influence of smaller longitudinal diffusion which overcompensated the dilution factor. This combined insight from effects (i), (ii), (iii), and (iv) enables us to define a normalization factor by which amplitudes obtained at other flow rates would have to be multiplied to be comparable on the expected response at 1.4 mL/min (eq 5).

5 $$f_{\left(i\right)}=\frac{\mathrm{Peak}\;\mathrm{amplitude}\;\mathrm{at}\;1.4}{\mathrm{Peak}\;\mathrm{amplitude}\;\mathrm{at}\;i}$$

**Figure 5:**
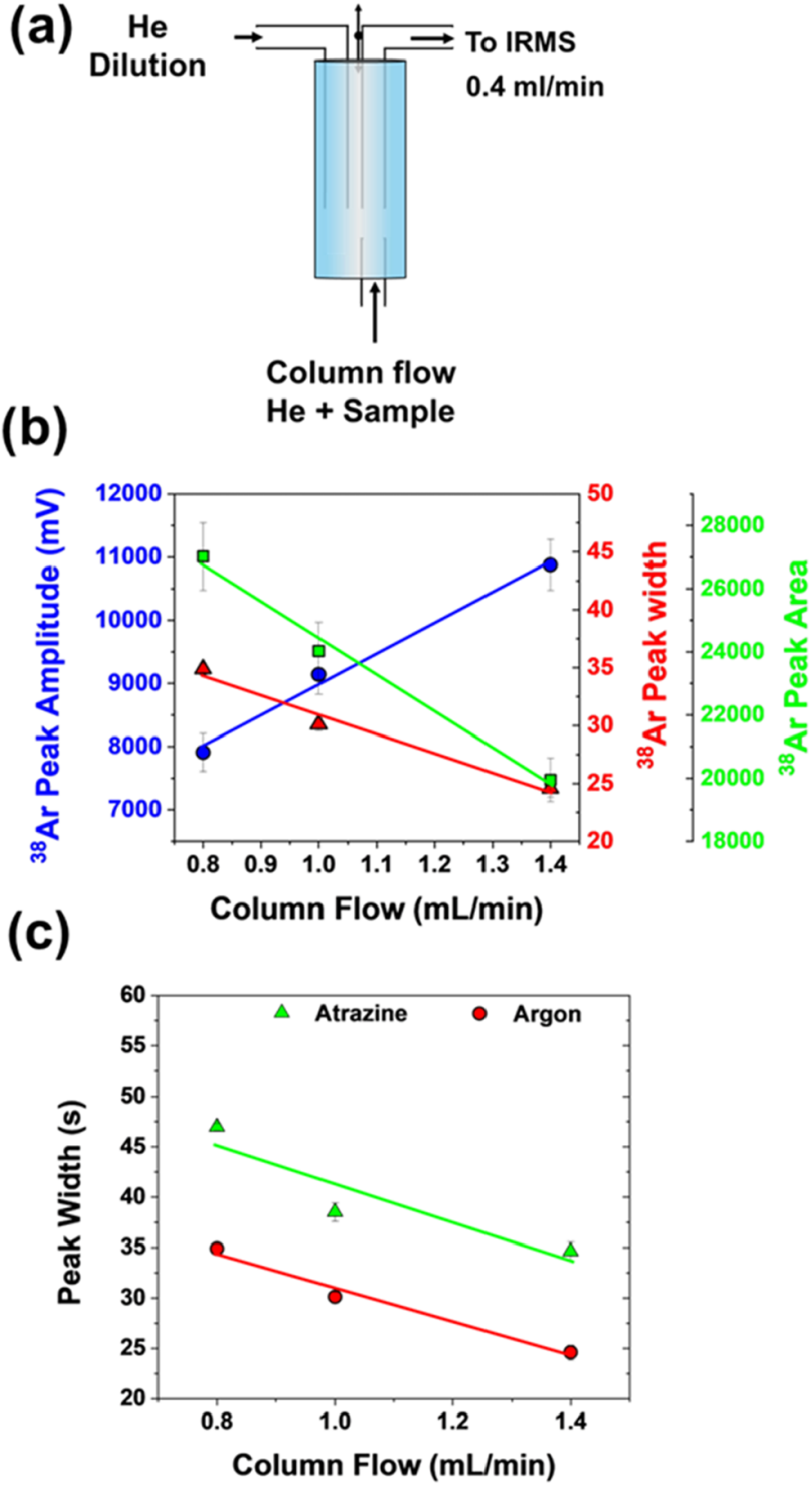
(a) Schematic of the Conflo open split before the IRMS and the effect of column flow on the response of argon as the inert analyte: (b) peak amplitude (blue circle) vs area (green square) and width (red rectangle); (c) peak width for atrazine (4 nmol C on-column) and argon.

Correspondingly, the calculated normalization factors for a flow of 1.4 mL/min is *f*_1.4_ = 1.0, whereas the factors for flows of 0.8 and 1.0 mL/min were *f*_0.8_ = 1.4, and *f*_1.0_ = 1.2.

Figure 5c demonstrates that the peak widths of atrazine and argon follow the same trend as the flow rate increases. This indicates that longitudinal diffusion in capillaries of the interface (effect (ii)) is the dominant contributor to peak broadening rather than effects in the GC column itself. In the next section, these normalization factors are used to assess the influence of conversion efficiency (effect (iv)) separately.

#### Conversion Effect

Atrazine has a more complex chemical structure than most other target compounds in CSIA, distinguished by the aromatic triazine ring and substitution by a chlorine atom. Accordingly, previous work has shown that conversion of atrazine is more difficult to accomplish than that of other substances, with a potential for incomplete conversion and systematic isotope fractionation.^7^ When measured using a commercial NiO/CuO/Pt reactor operated at the recommended temperature of 940 °C, the accuracy and precision of the results were reported to be prohibitive for reliable carbon isotope analysis.^23^ Improved accuracy of ± 0.7, in contrast, was reported with a self-made reactor containing NiO wires and operating at 1150 °C.^23^ Here, we tested the performance of the Ni-WCRs with atrazine as a benchmark for molecular structures that are particularly recalcitrant to oxidation. When atrazine was analyzed on Ni-WCRs, Ni-0.20ID-Alumina, and Ni-0.30ID-Quartz under the flow conditions that gave the greatest amplitude in Figure 5 (1.4 mL/min), accurate δ^13^C data were obtained (Figure S3). However, the linearity for carbon and nitrogen measurements with Ni-0.20ID-Alumina showed that an increase in sample size caused the δ values to shift toward more positive values indicating incomplete conversion of atrazine. This shift was particularly pronounced in nitrogen isotope analyses (Figure 6a vs [Fig fig6]d). In response, we decreased the column flow to allow for increased residence times in the reactor, and assessed the resultant linearity. At a decreased flow rate, isotope values became, indeed, less amount-dependent and more accurate (red circles in Figure 6a and 6d). The underlying reason became apparent when plotting amplitudes against the nominal amount of substance on-column (Figure 6b and 6e) and when correcting these amplitudes subsequently for the effect of dilution and peak height using the normalization factors from eq 5 (Figure 6c and 6f). In discrepancy with the Ar measurements, the decrease in flow did not decrease the uncorrected amplitude in Figure 6b and 6e indicating that a countertrend of improved conversion efficiency must have been at work. Interestingly, this trend would not have been picked up otherwise because *m*/*z* 30 traces were unsuspicious (see Figure S4). Figure 6c and 6f explicitly confirms this conclusion. The plot reveals that normalized amplitudes actually increased at lower flow rates, a phenomenon that became more pronounced at higher concentrations where larger sample amounts had to be converted. Notably, similar trends were observed for caffeine (Figure S5), the conversion of which was also improved at lower flow, even though δ values remained unaffected.

**Figure 6:**
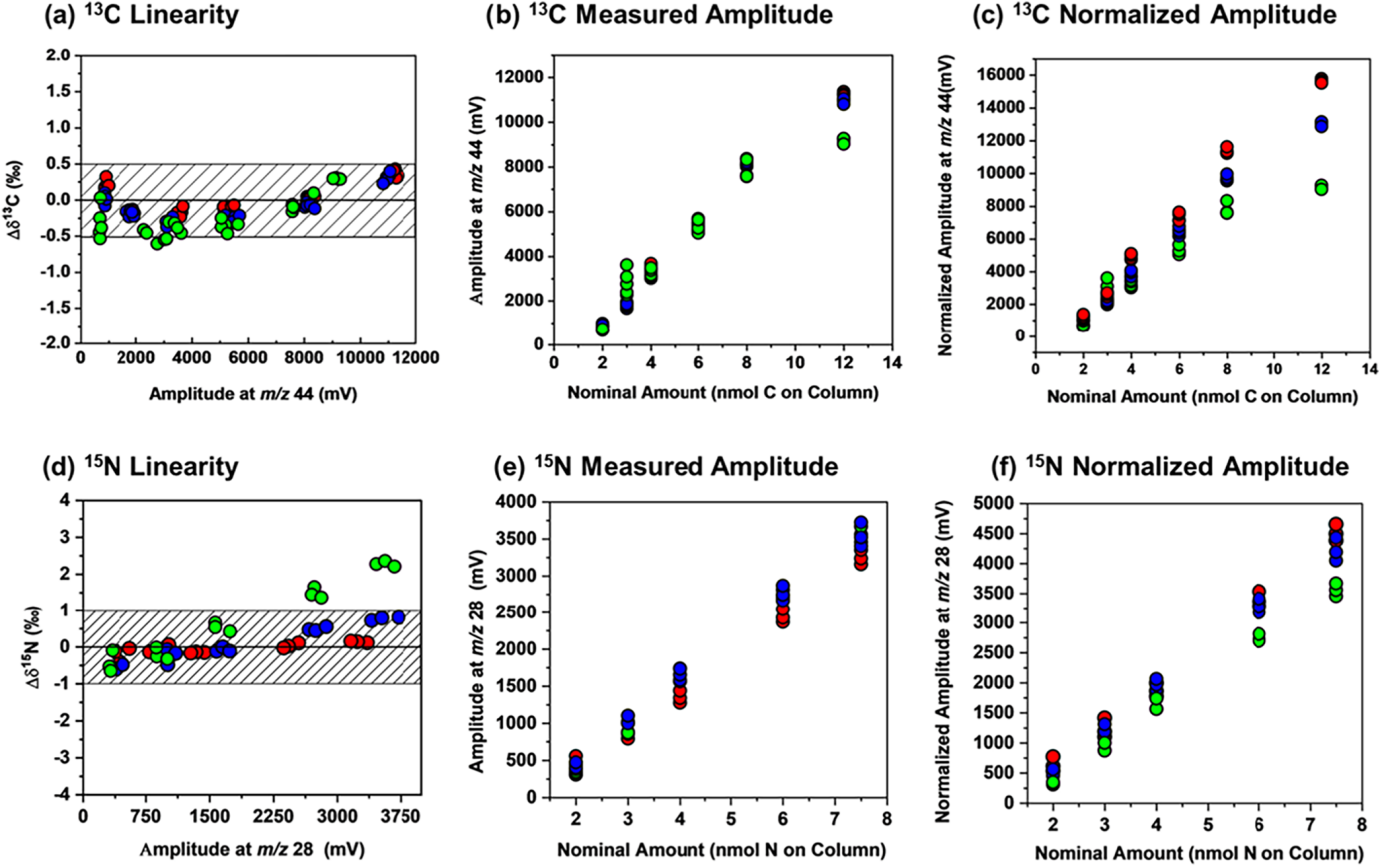
Amount-dependency in compound-specific isotope analysis of atrazine using a Ni-0.20ID-Alumina reactor. (a) Δδ^13^C versus peak amplitudes of *m*/*z* 44 for different column flow rates (green: 1.4 mL/min, blue:1.0 mL/min and red: 0.8 mL/min); (b) the corresponding peak amplitudes of *m*/*z* 44 versus the nominal amount carbon on-column; (c) the same plot as in panel (b), only with normalized peak amplitudes of *m*/*z* 44 using the normalization factor of eq 5; (d) Δδ^15^N versus peak amplitudes of *m*/*z* 28 for different column flow rates; (e) the corresponding peak amplitudes of *m*/*z* 28 versus the nominal amount nitrogen on-column; (f) the same plot as in panel e, only with normalized peak amplitudes of *m*/*z* 28 using the normalization factor of eq 5.

### Conventional versus Narrow-Bore Interface

With conventional GC columns, lower volumetric flow rates would be disadvantageous because they translate into low linear flow rates causing peak broadening (Figure 5c). When employing narrow-bore capillary GC columns, however, optimal linear flow velocities will translate into volumetric carrier gas flow rates that are well compatible with the optimum (<1.0 mL/min) observed in Figure 6. In addition to enhancing the residence time in the reactor, they will also minimize losses in the open split (see Figure 5a). Hence, the integration of fast-GC with WCRs is expected to sharpen peaks and improve sensitivity for multiple reasons.

To assess the N-WCR’s ability to minimize peak broadening compared to commercial reactors, we conducted a proof-of-principle test by injecting an *n*-alkane mix, specifically *n*-C_11_ to *n*-C_15_, into two GC setups: one employing narrow-bore capillaries including Ni-0.20ID-Alumina (Reactor 3), and the other featuring a commercial GC-Isolink interface, both operated with the same GC method described before but with the initial temperature 60 °C. The results, as depicted in Figure 7a, show notably higher peaks for the narrow-bore system when 130 pmol C on-column was measured, demonstrating its superior performance compared to the GC-Isolink system where the peaks were not detected for this amount. Figure 7b further illustrates the peak shape for combusted *n*-C_11_ measured by the narrow-bore system and its equivalent height peak (1.3 nmol on C on-column) by Isolink. It shows that the peaks in the narrow-bore system have a full width half-maximum that was more than 30% smaller compared to the commercial GC-Isolink system. This advancement highlights the benefits of the optimized reactor design and of the configuration for enhanced peak resolution and sensitivity. The mean measured δ^13^C values, the standard deviation of replicate measurements (SD), and the number of replicates (n) summarized in Table 2 show excellent agreement between isotope values measured in narrow-bore vs GC-Isolink reactors.

**Figure 7:**
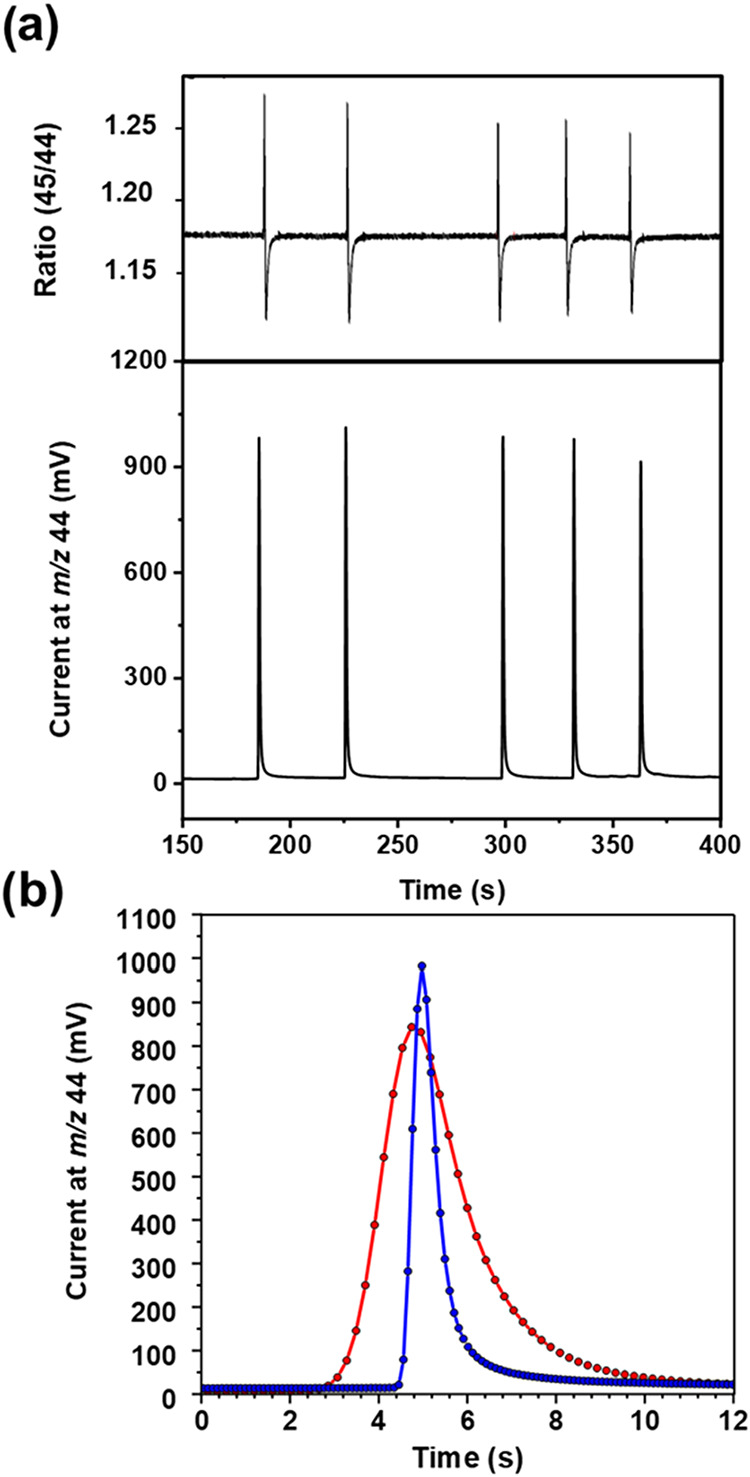
(a) Chromatogram of *n*-alkanes measured by our narrow-bore (a) CO_2_ peaks generated by combusting 100 pmol carbon on-column of each *n*-C_11_ to *n*-C_15_ (bottom) with the isotope ratio trace (^13^C/^12^C) (top). The same amount on-column is not detected in the case of the GC-Isolink system. (b) Peak shapes of combusted *n*-C_11_ measured by our narrow-bore GC-C-IRMS (blue) vs 1 nmol C on-column measured by the commercial GC-Isolink system. Full width half-maximum (fwhm) for the peak eluting from the GC-Isolink system was 2.2 s, whereas the fwhm of peaks from the narrow-bore system was 0.61 s.

**Table 2:** Comparison of Isotopic Results from Narrow-Bore and Isolink Systems for Five *n*-Alkane Chain Lengths (*n*-C_11_ to *n*-C_15_)

	narrow-bore			GC-Isolink		
*n*-alkane	mean δ^13^C (‰)	SD (‰)	*n*	mean δ^13^C (‰)	SD (‰)	*n*
*n*-C_11_	–28.0	0.3	4	–28.0	0.1	4
*n*-C_12_	–31.5	0.3	4	–31.5	0.1	4
*n*-C13	–33.2	0.4	4	–32.8	0.1	4
*n*-C14	–30.1	0.2	4	–29.7	0.1	4
*n*-C15	–30.8	0.1	4	–31.2	0.1	4

## Conclusions

The combustion of organic target compounds in a continuous helium flow within a miniature reactor tube is a crucial step in compound-specific isotope analysis utilizing GC-C-IRMS. While GC-C-IRMS has facilitated a surge of new research directions across disciplines, many applications are still out of reach because they require sharper and higher peaks for better sensitivity and resolution. To drive this development with a step change in the miniaturization and robustness of conversion reactors, we designed and developed nickel wall-coated microreactors through electroless plating. Instead of inserting a wire into a narrow-bore capillary, we deposited a thin nickel layer onto the inner wall of a narrow-bore capillary tube. This innovative design drastically reduced the reactor volume while maintaining the catalytic surface area and ensuring robustness and longevity with several hundred measurements per reactor. The reactors’ performance for isotope analysis was validated with heterocyclic aromatic compounds as notoriously challenging target compounds for complete conversion. Results for caffeine, atrazine, and 1H-benzotriazole demonstrated exceptional accuracy and reliability in both carbon and nitrogen isotope analysis. Measurements of atrazine highlight, in addition, the importance of sufficiently long residence times in the reactor, which can advantageously be reached with low volumetric flow rates in narrow separation columns of fast gas chromatography. In the overarching aim to improve sensitivity and peak resolution, our proof-of-principle study demonstrated a 10-fold increase in sensitivity and a 2-fold improvement in peak resolution compared to the current commercial setup. This study therefore lays the foundations for much-needed developments in CSIA for which peak width is critical, specifically, fast GC and comprehensive two-dimensional gas chromatography-IRMS (GCxGC-C-IRMS).

## Supplementary Material



## Notes

Detailed characterization of the Ni coating used in the reactor system, isotope ratio traces obtained during analysis, complete results for isotope analysis of atrazine using the two reactor configurations, evaluation of the baseline behavior at *m*/*z* 30 under different column flow conditions, and assessment of the effect of column flow on the compound-specific isotope analysis (CSIA) of caffeine (PDF)
