# Supplementary material for: Nickel Wall-coated Microreactors for Enhanced Sensitivity and Peak Resolution in Compound-specific Carbon and Nitrogen Isotope Analysis
Source: Anal Chem. 2026 May 18;98(21):15565–75. doi: 10.1021/acs.analchem.6c00746 (PMC13234822; doi:10.1021/acs.analchem.6c00746)
Supplement: Supplementary file 1 [file ac6c00746_si_001.pdf]

Supplementary Information

Nickel Wall-coated Microreactors for Enhanced Sensitivity and Peak Resolution in Compound-specific Carbon and Nitrogen Isotope Analysis

*Habib Al-Ghoul and Martin Elsner**

Technical University of Munich, TUM School of Natural Sciences, Department of Chemistry, Chair of Analytical Chemistry and Water Chemistry, Lichtenbergstraße 4, 85748 Garching, Germany

*Email: m.elsner@tum.de. Phone: +49 (89) 289-54500.

Contents

S1 Characterization of the Ni Coating S-2
S2 Isotope ratio trace S-4
S3 Results for Isotope Analysis of Atrazine on the Two Reactors S-5
S4 30 m/z Baseline at different column flow S-5
S5 Effect of Column Flow on CSIA of Caffeine S-6

S1 Characterization of the Ni Coating

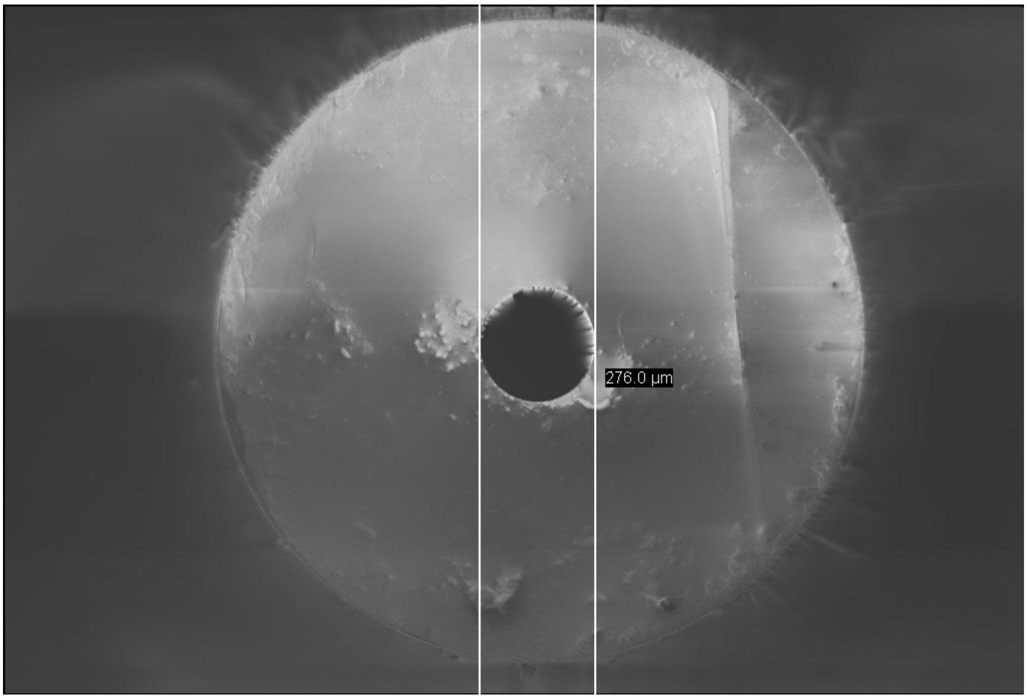

Figure S1: Cross-sectional SEM-EDX images and optical microscope of Ni-0.28ID Quartz capillary microtubes.

Table S1:

Table S1: The data from ICP-MS and SEM measurements of Ni-0.28ID Quartz capillary microtubes (experimental replicates)

	Deposited mass from ICP-MS (mg/cm)	Measured Tube ID (D_i) from SEM (μm)	Measured layer thickness from SEM (μm)
	0.079	276	1.455
	0.089	275	1.287
	0.089	279	1.210
	0.11	277	1.164
	0.12	272	1.026
	0.084	275	1.266
	0.11	276	1.220
	0.13	277	-
Average	0.10	276	1.23

Calculation of the theoretical Nickel Tube Wall Thickness (t)

Given:

- Outer cross section of the inner diameter (D_i) (Average) = 0.276 mm
(information from microcopic images summarized in the second column of Table S1)
→ A diameter of 0.276 mm corresponds to a radius of 0.138 mm

- Mass of Ni deposited on 1 cm: approximately 0.1 mg = 0.0001 g
(Information from quantification by ICP-MS summarized in the first column of Table S1)

- Length considered for the deposition of Ni (first column of Table S1): 1 cm = 10 mm

- Density of Nickel = 8.90 g/cm³ = 0.00890 g/mm³

Formula:

Assuming that the Ni deposite is a hollow cylinder (Ni-wall tube) inside the reactor tube. The volume (V) of this wall tube is:

$$V = \pi L(r^2_o - r^2_i) \quad (1)$$

Where,

r_o is the outer readious of the Ni-wall tube and it is equal to half of inner diamter of the reactor tube (D_i).

r_i is the inner readious of the Ni-wall tube ($r_i = r_o - t$), where t is the tube wall thickness.

So

$$V = \pi L(r^2_o - (r_o -t)^2) \quad (2)$$

$$V = \pi L(r^2_o - (r^2_o - 2rt+t^2) \quad (3)$$

$$V = \pi L(2r_o t-t^2) \quad (4)$$

Relate volume to mass (m)

$$m = \rho \cdot V \quad (5)$$

$$m = \rho\pi L(2r_o t - t^2) \quad (6)$$

Where (m) is the mass of nickel, (ρ) is its density

Substitute values:

$0.0001 = 0.00890 \times \pi \times 10 \times (0.276t - t^2)$

$0.0001 = 0.2795 \times (0.276t - t^2)$

$0.0003578 \approx 0.276t - t^2$

Quadratic equation:

$t^2 - 0.276t + 0.0003578 = 0$

Solve using quadratic formula:

$t = (0.276 \pm \sqrt{(0.276^2 - 4 \times 1 \times 0.0003578)}) / 2$

$t = (0.276 \pm \sqrt{0.0747448}) / 2$

$t = (0.276 \pm 0.2734) / 2$

Solutions:

- $t_1 = (0.276 - 0.2734) / 2 = 0.0013$ mm
- $t_2 = (0.276 + 0.2734) / 2 = 0.2747$ mm → not plausible

Thus,

The calculated coated wall thickness is approximately 0.0013 mm or 1.3 µm, which is in excellent agreement with the measured layer thickness from scanning electron microscopy images summarized in the third column of Table 1.

S2 Isotope ratio trace

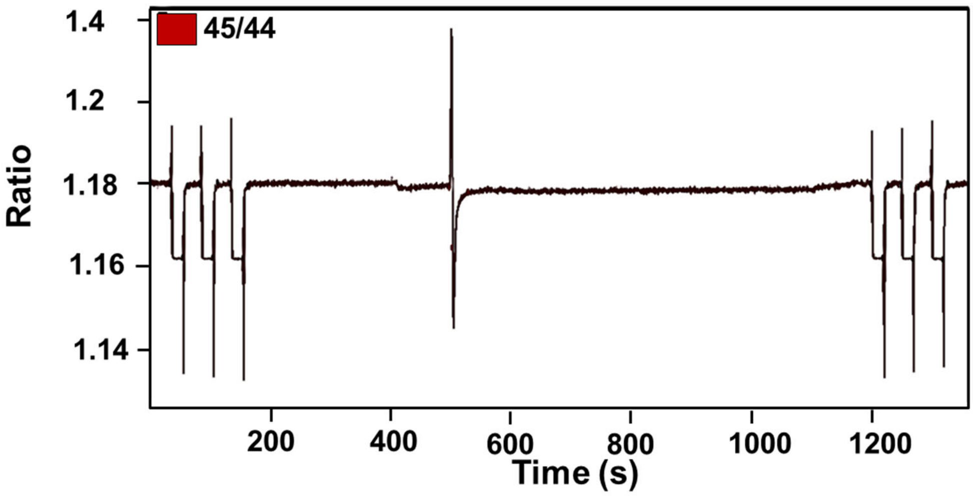

Figure S2: The isotope ratio trace ($^{13}C/^{12}C$) for 3nmol C on column caffeine analysis.

S3 Results for Isotope Analysis of Atrazine on the Two Reactors

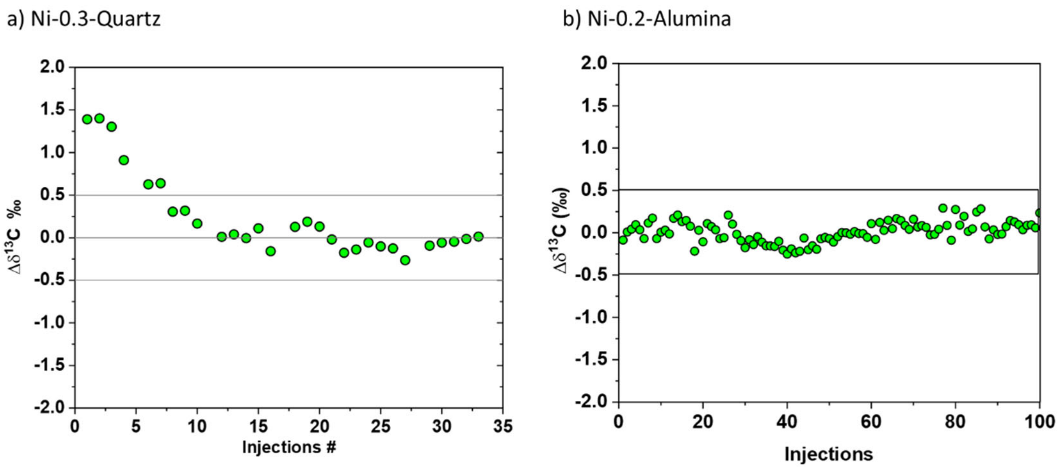

Figure S3: $\Delta\delta^{13}$C values of an atrazine standard using a) Ni-0.3-Quartz and b) Ni-0.2-Alumina.

S4 30 m/z Baseline at different column flow

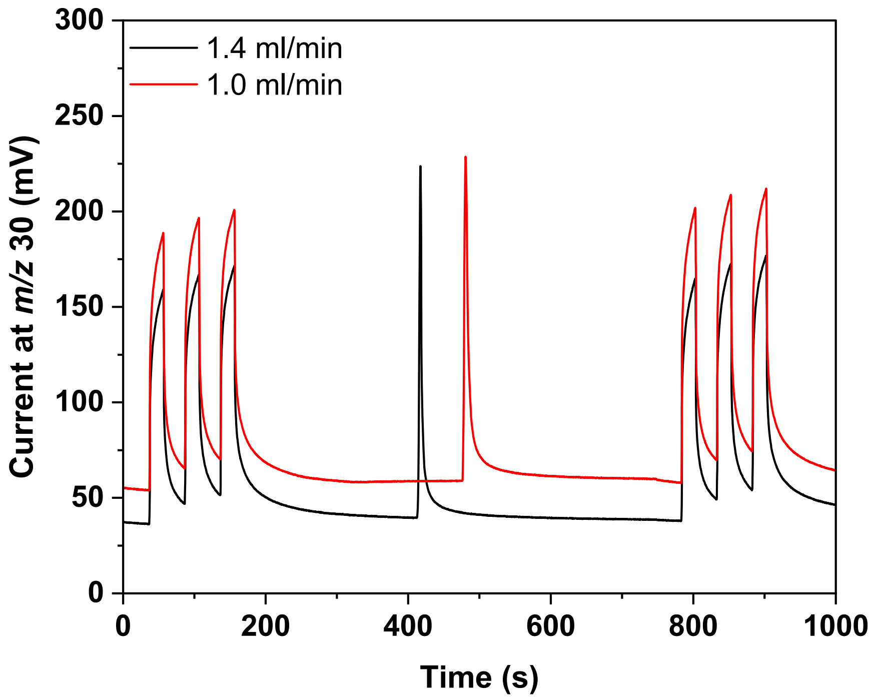

Figure S4: 30 m/z chromatogram for atrazine ^{15}N measurement at different column flow (ml/min), 1.0 (red) and 1.4 (black).

S5 Effect of Column Flow on CSIA of Caffeine

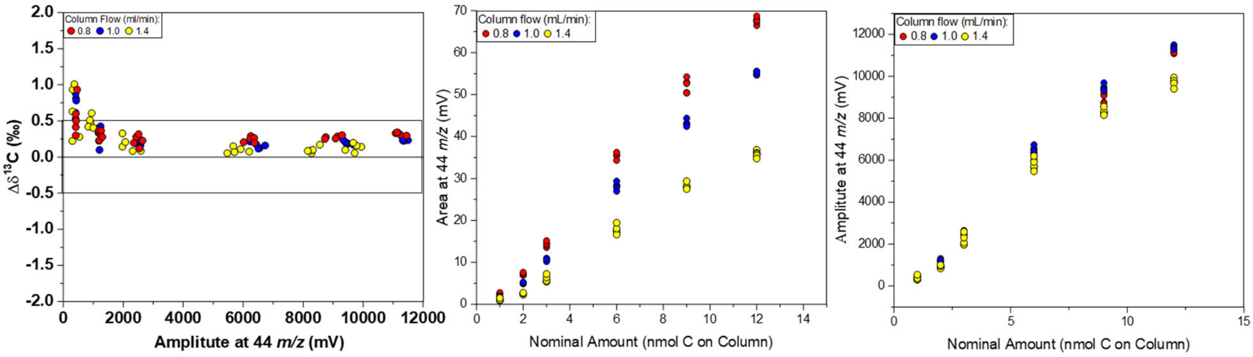

Figure S5: Compound-specific isotope analysis of caffeine using a Ni-0.2ID-Alumina reactor. a) $\Delta\delta^{13}C$ for peak amplitudes of m/z 44 of injected carbon at different column flow rates. b) Area of m/z 44 corresponding to the nominal amount of injected carbon. c) Peak amplitudes of m/z 44 corresponding to the nominal amount of injected carbon.
